# Risk factors and prognostic analysis of right ventricular dysfunction after lung resection for NSCLC

**DOI:** 10.3389/fonc.2024.1371594

**Published:** 2024-06-19

**Authors:** Xilun Tan, Jing Tao, Qin Zhang, Xiang Li, Jia Wang, Hao Song, Yanni Zhou, Sihan Wang, Jun Cheng, Ming Wang

**Affiliations:** ^1^ Chongqing Medical University, Chongqing, China; ^2^ Department of Cardiovascular Medicine, Chongqing Hospital of Traditional Chinese Medicine, Chongqing, China; ^3^ Department of Oncology, Chongqing Hospital of Traditional Chinese Medicine, Chongqing, China; ^4^ Department of Radiology, Chongqing Hospital of Traditional Chinese Medicine, Chongqing, China; ^5^ Department of Emergency, Wangjing Hospital of China Academy of Chinese Medical Sciences, Beijing, China; ^6^ Chongqing Shapingba Hospital of Chinese Medicine, Chongqing, China; ^7^ Chongqing College of Traditional Chinese Medicine, Chongqing, China

**Keywords:** non-small cell lung cancer, lung resection, right ventricular dysfunction, risk factors, prognostic analysis

## Abstract

**Objectives:**

Lung cancer is the leading cause of cancer death, and 80–85% of all lung cancer cases are non-small cell lung cancer (NSCLC). Surgical resection is the standard treatment for early-stage NSCLC. However, lung resection, a surgical procedure, can result in complications and increased mortality. Recent studies have shown a significant correlation between complications after lung resection and right ventricular dysfunction.

**Methods:**

Transthoracic echocardiography-derived right ventricular-pulmonary artery coupling (RV-PAC) was utilized to assess right ventricular function in these patients. Multivariate logistic regression analysis was also conducted to assess risk factors independently associated with RV-PA uncoupling. The 3- and 5-year cumulative survival rates were estimated with Kaplan-Meier curves, and differences between groups were analyzed using the Mantel-Cox log-rank test.

**Results:**

RV-PA uncoupling was defined as a TAPSE/PASP value < 0.67 mm/mm Hg according to spline analysis. The results of multivariable logistic regression analysis indicated that diabetes is an independent risk factor for right ventricular dysfunction after lung resection in patients with NSCLC. Kaplan-Meier analysis revealed a significant decrease in the survival rate of patients with RV-PA uncoupling at both the 3-year follow-up (73% vs 40%, p < 0.001) and 5-year follow-up (64% vs 37%, p < 0.001).

**Conclusions:**

After lung resection for NSCLC, the patient’s right ventricular function predicts prognosis. Patients with right ventricular dysfunction, particularly those with diabetes mellitus, have a worse prognosis. It is crucial to actively prevent and correct risk factors to reduce the mortality rate in these patients.

## 1 Introduction

Lung cancer is a prevalent malignant tumor worldwide and has the highest mortality rate among all malignant tumors (1). Non-small cell lung cancer (NSCLC) accounts for 80–85% of all lung cancer cases (2). Surgical resection is the standard treatment for early-stage NSCLC and provides the best chance of cure (2). However, lung resection, which is a common surgical procedure, can lead to complications during the perioperative period, potentially increasing mortality rates (3, 4). Previous studies have suggested that postoperative complications after lung resection are related to the deterioration of pulmonary function. However, recent studies have indicated that these complications are primarily associated with cardiac dysfunction, particularly right ventricular dysfunction (5–7). Therefore, it is crucial to assess right ventricular function in this patient group.

The RV is a thin-walled crescent-shaped structure coupled to systemic venous return on one side and to the pulmonary circulation on the other. Its main function is to regulate the flow of blood returning from the veins into a consistent output per beat, which is then pumped into the low-resistance pulmonary circulation. This ensures that the left ventricle receives sufficient blood volume and maintains adequate cardiac output to meet the body’s perfusion needs (8). However, when patients experience right ventricular dysfunction, the RV rapidly transitions from compensation to decompensation. This creates a detrimental cycle that significantly impacts hemodynamics, leading to poor blood flow throughout the body, reduced perfusion pressure in organs and tissues, and, ultimately, a notable increase in mortality (9, 10).

In recent years, the unique structure and function of the right ventricle have limited the availability of methods for assessing its function. However, advancements in medical imaging technology have led to an increasing number of methods for evaluating right ventricular function. This approach offers the potential for improved research in this area (11, 12). Previous studies have established that right ventricular dysfunction is a significant risk factor for increased mortality following lung resection in patients with NSCLC. Therefore, it is crucial to actively assess right ventricular function and identify the risk factors that contribute to RV dysfunction in these patients. This proactive approach is vital for preventing and correcting right ventricular dysfunction and reducing perioperative mortality.

This study aimed to retrospectively analyze the clinical characteristics of right ventricular dysfunction that occurs after lung resection in patients with NSCLC, summarize the risk factors associated with right ventricular dysfunction in these patients, and analyze the significance of right ventricular function in predicting the prognosis of these patients.

## 2 Materials and methods

### 2.1 Patients

**Figure 1:**
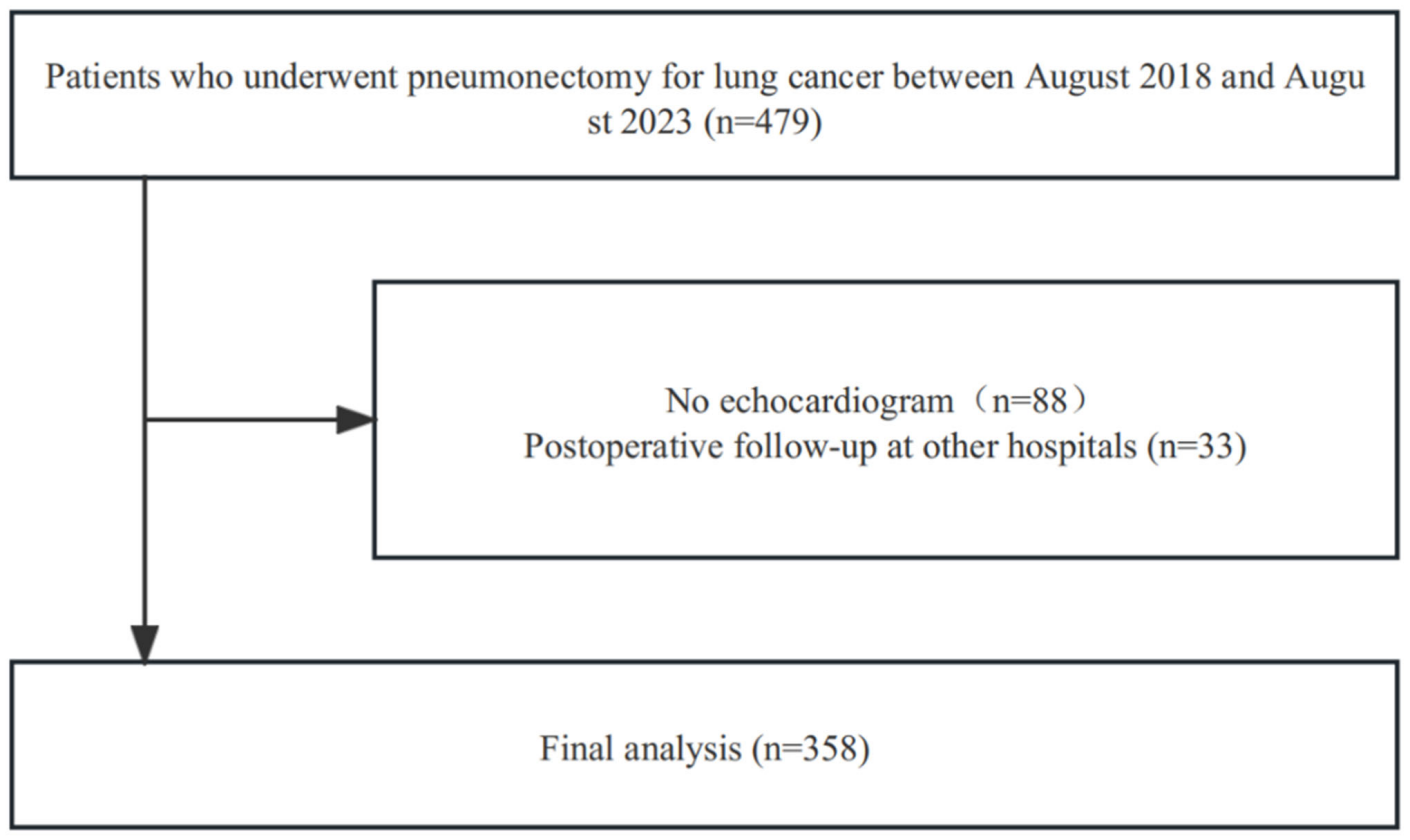
Patient’s flowchart.

This retrospective study included 479 patients who were admitted to Chongqing Traditional Chinese Medicine Hospital from August 2018 to August 2023 after undergoing lung resection for non-small cell lung cancer (NSCLC). However, 121 of these patients were excluded from the study for the following reasons (**Figure 1**): 88 had no incomplete transthoracic echocardiography data for assessing right ventricular function, and 33 were followed up in other hospitals. Overall, 358 patients met the inclusion criteria and were included in the study. All patients underwent transthoracic echocardiography before lung resection, and they had no preoperative right ventricular dysfunction. This retrospective study adhered to the Declaration of Helsinki and relevant ethical policies in China. The Institutional Review Board and Ethics Committee of Chongqing Hospital of Traditional Chinese Medicine approved this study. The requirement for patient consent was waived because of the retrospective study design.

### 2.2 Measurements

The analysis included patient demographic characteristics, perioperative characteristics, and echocardiographic data. Each patient underwent transthoracic echocardiography within 30 days of lung resection. Transthoracic echocardiographic data were acquired with patients at rest in the left lateral decubitus position using available ultrasound systems (Vivid 7, E9, and E95 systems; GE-Vingmed) equipped with 3.5 MHz or M5S transducers. Digital images were stored on disks for offline analysis using EchoPAC software (GE-Vingmed Ultrasound). The left ventricular ejection fraction (LVEF) was calculated using the biplane Simpson method. RV systolic function was estimated by measuring the tricuspid annulus plane systolic excursion (TAPSE) on M-mode recordings of the lateral tricuspid annulus. Pulmonary artery systolic pressure (PASP) was estimated by measuring the peak velocity of the tricuspid regurgitation (TR) jet and applying the simplified Bernoulli equation, with the addition of mean right atrial pressure. The mean right atrial pressure was derived based on the inferior vena cava diameter and collapsibility during inspiration. Right ventricular function was assessed in this study using right ventricular-pulmonary artery coupling (RV-PAC). RV-PA coupling was estimated noninvasively by calculating the ratio between two standard echocardiographic measurements: the TAPSE and PASP. RV-PA coupling refers to matching between right ventricular function and afterload and is crucial for assessing the severity of clinical disease and predicting outcomes (13, 14). Furthermore, recent studies have demonstrated that the TAPSE/PASP is the only echocardiographic index that is independently associated with the gold standard invasive measurement of RV-PA coupling (15).

The primary endpoint of the study was all-cause mortality. All patients were followed until the occurrence of the primary endpoint. Survival time was defined as the time from lung resection to the endpoint or the end of follow-up.

### 2.3 Statistical methods

The statistical analyses were performed using SPSS version 25.0 (SPSS Inc, IBM Corp) and in R environment 3.6.4 (R Foundation for Statistical Computing). Categorical variables are expressed as numbers and percentages. For continuous variables, adherence to a normal distribution was verified through visual assessment, comparing a histogram of the sample data to a normal probability curve. Normally distributed continuous variables are presented as the mean ± standard deviation, while variables that are non-normally distributed are presented as the median and interquartile range. To assess the hazard ratio (HR) change for all-cause mortality across a range of TAPSE/PASP values at baseline, a spline curve analysis was performed. Based on spline analysis, TAPSE/PASP defined the cut-off value for RV-PA uncoupling as chosen based on a significant increase in mortality. Differences between RV-PA coupling and uncoupling were analyzed using the unpaired Student t-test for normally distributed continuous variables, the Mann-Whitney U test for non-normally distributed continuous variables, and Pearson’s chi-square test for categorical variables. Multivariate logistic regression analyses were included if p values were < 0.05 in univariate logistic regression analyses. Risk factors independently associated with RV-PA uncoupling were assessed using multivariate logistic regression analysis. The 3- and 5-year cumulative survival rates were estimated with Kaplan-Meier curves, and differences between groups were analyzed using the Mantel-Cox log-rank test. A multivariate Cox proportional hazard regression analysis was also conducted to assess the clinical and echocardiographic features that were independently associated with all-cause mortality. Possible confounders with p values < 0.05 in the univariate analysis were included in the multivariate Cox regression analysis. HRs and 95% confidence intervals (CIs) were calculated. Two-sided p values < 0.05 were considered to indicate statistical significance.

## 3 Results

### 3.1 Spline curve for all-cause mortality according to TAPSE/PASP ratio

**Figure 2:**
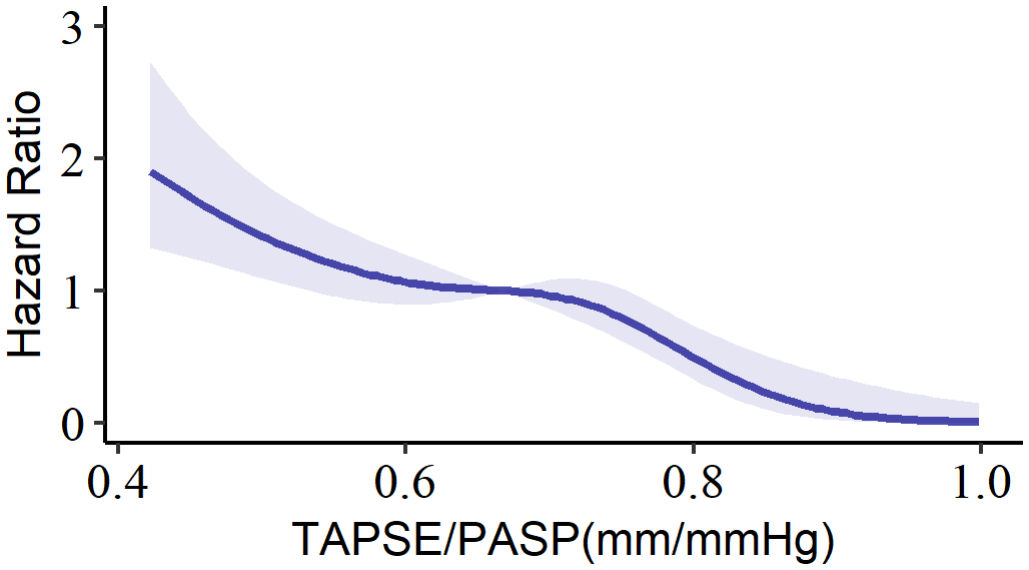
Spline curve for all-cause mortality according to TAPSE/PASP ratio. The curve represents the hazard ratio change for all-cause mortality with overlaid 95% confidence intervals (light-blue) across a range of TAPSE/PASP ratio. TAPSE/PASP, Tricuspid annular plane systolic excursion/pulmonary artery systolic pressure ratio.

A total of 358 patients were enrolled, with a median age of 65 years (interquartile range, 61 to 71), and 62.6% of the participants were male. To investigate the relationship between the TAPSE/PASP ratio and all-cause mortality, a spline analysis was performed (**Figure 2**). The hazard ratio (HR) for all-cause mortality showed an initial slow increase but significantly increased as the TAPSE/PASP ratio decreased (< 0.67 mm/mm Hg). Based on this analysis, TAPSE/PASP values < 0.67 mm/mm Hg were used to define RV-PA uncoupling and categorize the population.

### 3.2 Patient characteristics

After lung resection for NSCLC, 193 patients (53%) developed RV-PA uncoupling. In comparison to those with RV-PA coupling, those with RV-PA uncoupling were older and had a greater incidence of heart failure and diabetes mellitus. Patients with RV-PA uncoupling used radiotherapy more and had higher high-sensitivity troponin T. (**Table 1**). Additionally, RV-PA uncoupling was associated with more patients with pathologic staging of adenocarcinoma and adenosquamous carcinoma, fewer patients with clinical staging of stages I and II, and more patients with stage IV disease (**Table 2**). Furthermore, RV-PA uncoupling resulted in a greater tricuspid maximal regurgitant velocity and PASP and a lower TAPSE than RV-PA coupling did (**Table 3**).

**Table 1:** Clinical and demographic characteristics.

Variable	RV−PA coupling (TAPSE/PASP ≥ 0.67) (n = 165)	RV−PA uncoupling (TAPSE/PASP < 0.67) (n = 193)	p value
Demographic			
Age (year)	64.1 ± 8.4	66.0 ± 7.8	0.031
Sex (male)	100 (61%)	124 (64%)	0.478
Current or former smoker	88 (53%)	105 (54%)	0.915
Cardiovascular comorbidity			
Hypertension	43 (26%)	57 (30%)	0.465
Hypercholesterolemia	18 (11%)	17 (9%)	0.505
Coronary artery disease	8 (5%)	18 (9%)	0.104
Heart failure	7 (4%)	20 (10%)	0.029
Atrial fibrillation	3 (2%)	4 (2%)	0.862
Cerebral infarction	18 (11%)	34 (18%)	0.066
Chemotherapy			
platinum-based drugs and antimetabolites	48 (29%)	74 (38%)	0.66
platinum-based drugs and antimicrotubule drugs	61 (37%)	70 (36%)	0.891
Targeted Drugs	7 (4%)	10 (5%)	0.677
Others			
COPD	8 (5%)	14 (7%)	0.384
Diabetes mellitus	23 (14%)	49 (25%)	0.007
Chronic renal failure	1 (1%)	7 (4%)	0.117
Radiotherapy	16 (1064%)	42 (22%)	0.002
Heart rate (bpm)	86.8 ± 13.7	89.3 ± 16.0	0.146
B-type natriuretic peptide (pg/mL)	127.4 ± 169.6	270.0 ± 849.9	0.249
High-sensitivity troponin T (pg/mL)	7.1 ± 5.2	64.1 ± 49.6	0.043

Values are presented as mean ± standard deviation or n (%). COPD, chronic obstructive pulmonary disease.

**Table 2:** Perioperative characteristics.

Operative Variables	RV−PA coupling (TAPSE/PASP ≥ 0.67) (n = 165)	RV−PA uncoupling (TAPSE/PASP < 0.67) (n = 193)	p value
Pneumonectomy	2 (1%)	9 (5%)	0.059
Lobectomy			
Right upper lobe	59 (36%)	61 (32%)	0.407
Right middle lobe	8 (5%)	20 (10%)	0.053
Right lower lobe	37 (22%)	28 (15%)	0.053
Left upper lobe	43 (26%)	55 (28%)	0.606
Left lower lobe	30 (18%)	30 (16%)	0.505
Surgical approach (VATS)	156 (95%)	177 (92%)	0.294
Histological type			
Adenocarcinoma	130 (79%)	132 (68%)	0.027
Squamous carcinoma	27 (16%)	47 (24%)	0.063
Adenosquamous	1 (1%)	8 (4%)	0.042
Others	7 (4%)	5 (3%)	0.387
Clinical Stage			
I	57 (35%)	38 (20%)	0.002
II	23 (14%)	10 (5%)	0.004
III	17 (10%)	19 (10%)	0.886
IV	84 (51%)	145 (75%)	<0.001

Values are presented as n (%).VATS,video-assisted thoracic surgery.

**Table 3:** Echocardiographic characteristics.

Variable	RV−PA coupling (TAPSE/PASP ≥ 0.67) (n = 165)	RV−PA uncoupling (TAPSE/PASP < 0.67) (n = 193)	p value
Left ventricular end-diastolic diameter (mm)	43.8 ± 3.8	43.8 ± 4.0	0.905
Left ventricular end-systolic diameter (mm)	27.3 ± 3.2	27.4 ± 3.5	0.922
Interventricular septal thickness (mm)	10.3 + 1.9	10.2 ± 1.3	0.823
peak Tricuspid regurgitation velocity (cm/s)	228.7 ± 24.1	264.6 ± 28.8	<0.001
Maximal velocity of pulmonary valve (cm/s)	85.8 ± 15.2	86.3 ± 18.9	0.678
Left ventricular ejection fraction (%)	66.3 ± 5.1	65.9 ± 5.7	0.512
stroke volume (ml)	58.1 ± 10.5	57.3 ± 8.7	0.288
Tricuspid annular plane systolic excursion (mm)	20.8 ± 2.1	18.8 ± 2.3	<0.001
Pulmonary artery systolic pressure	23.6 ± 6.6	33.5 ± 6.8	<0.001

Values are presented as mean ± standard deviation.

### 3.3 Risk factors of right ventricular dysfunction after lung resection for NSCLC

**Tables 4**, **5** present univariate and multivariate logistic regression analyses of right ventricular dysfunction after lung resection for NSCLC patients. Univariate logistic regression analysis revealed significant associations between the following parameters and right ventricular dysfunction: age, heart failure status, diabetes status, use of radiotherapy, pathological classification as adenocarcinoma, clinical stage IV, and peak tricuspid regurgitation velocity. Multivariate logistic regression analysis revealed that diabetes mellitus was an independent risk factor for right ventricular dysfunction after lung resection for NSCLC.

**Table 4:** Univariable logistics regression analysis.

Variable	Univariate analysis Odds ratio (95% CI)	P value
Age	1.031 (1.004–1.058)	0.024
Sex (male)	1.168 (0.761–1.794)	0.478
Current or former smoker	1.044 (0.688–1.584)	0.839
Hypertension	1.189 (0.747–1.894)	0.466
Heart failure	2.609 (1.074–6.337)	0.034
Atrial fibrillation	1.143 (0.252–5.182)	0.863
COPD	1.535 (0.627–3.755)	0.348
Diabetes mellitus	2.116 (1.224–3.656)	0.007
platinum-based drugs and antimetabolites	1.516 (0.972–2.363)	0.066
platinum-based drugs and antimicrotubule drugs	0.970 (0.630–1.494)	0.891
Targeted Drugs	1.233 (0.459–3.316)	0.678
Radiotherapy	2.590 (1.395–4.809)	0.003
Adenocarcinoma	0.583 (0.360–0.942)	0.028
Squamous carcinoma	1.645 (0.971–2.788)	0.064
Adenosquamous	7.092 (0.878–57.306)	0.066
Clinical Stage IV	2.913 (1.863–4.554)	<0.001
peak Tricuspid regurgitation velocity	1.072 (1.054–1.090)	<0.001
Left ventricular ejection fraction (%)	0.987 (0.950–1.026)	0.511

Values are presented as median (quartile 1, quartile 3). COPD, chronic obstructive pulmonary disease.

**Table 5:** Multivariable logistics regression analysis.

Variable	Multivariate analysis Odds ratio (95% CI)	P value
Age	1.008 (0.973–1.045)	0.647
Heart failure	1.835 (0.590–5.706)	0.295
Diabetes mellitus	2.788 (1.371–5.669)	0.005
Radiotherapy	1.630 (0.767–3.460)	0.204
Adenocarcinoma	0.610 (0.325–1.142)	0.122
Clinical Stage IV	1.785 (0.966–3.301)	0.065
peak Tricuspid regurgitation velocity	1.071 (1.052–1.090)	0.168

Values are presented as median (quartile 1, quartile 3).

### 3.4 Prognostic analysis of right ventricular dysfunction after lung resection for NSCLC

**Figure 3:**
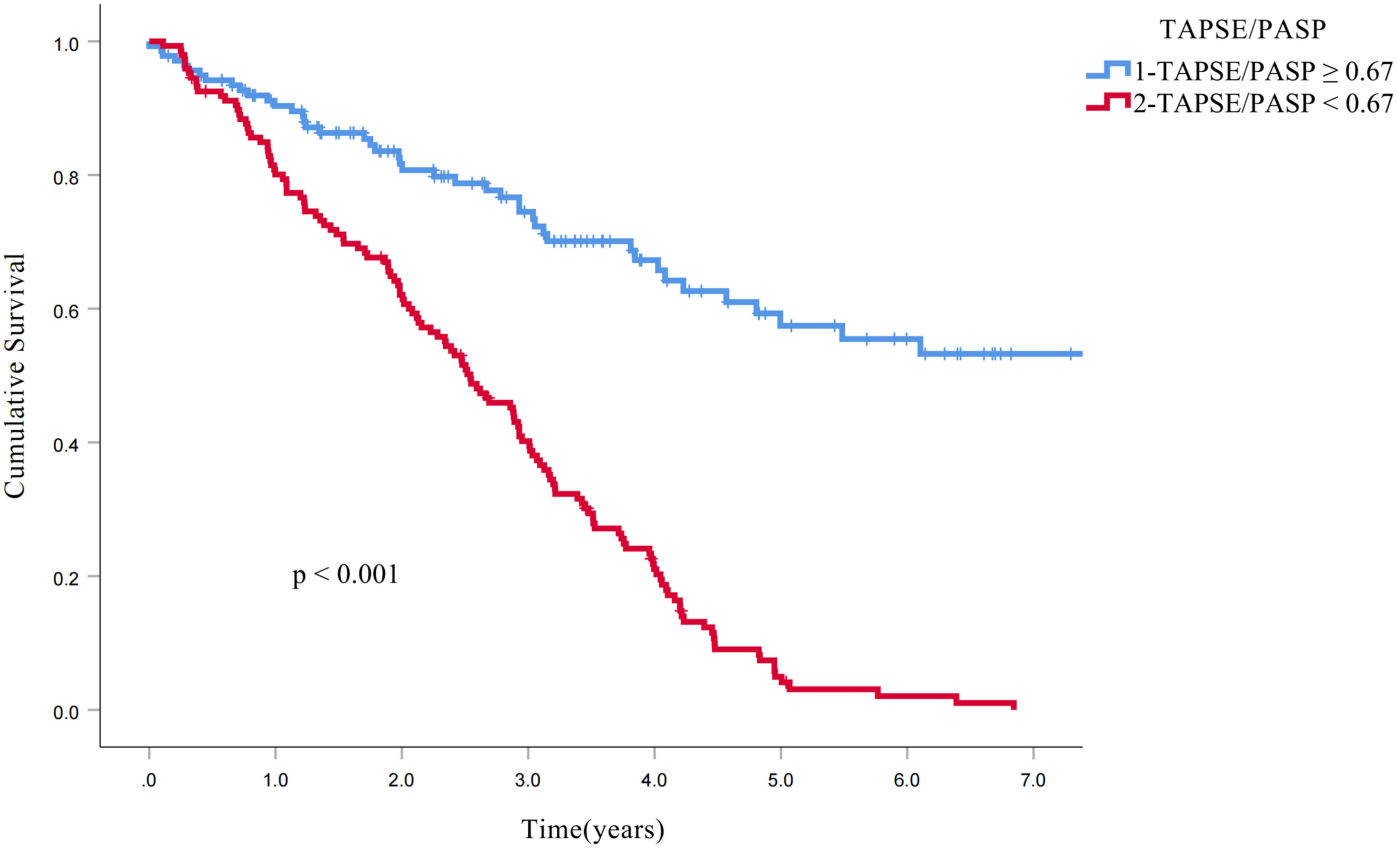
Kaplan−Meier curves for all-cause mortality. The Kaplan−Meier curves demonstrate the higher survival rates of patients with RV−PA coupling (TAPSE/PASP ratio: ≥0.67 mm/mm Hg, blue line) compared to those with RV−PA uncoupling (TAPSE/PASP ratio <0.67 mm/mm Hg, red line). RV-PA, Right ventricular − pulmonary arterial; TAPSE/PASP, Tricuspid annular plane systolic excursion/pulmonary artery systolic pressure ratio.

Kaplan-Meier analysis demonstrated significantly reduced survival in patients with RV-PA uncoupling at the 3-year follow-up (73% vs 40%, p < 0.001) and 5-year follow-up (64% vs 37%, p < 0.001; **Figure 3**). To further investigate the association between the TAPSE/PASP ratio and all-cause mortality, univariate and multivariate Cox proportional hazards models were constructed (**Tables 6**, **7**). The TAPSE/PASP was introduced as a categorical variable utilizing the threshold derived from spline curve analysis (0.67 mm/mm Hg). Univariate Cox regression analysis demonstrated significant associations between the following parameters and the risk of all-cause mortality: male sex, smoking history, heart failure, use of radiotherapy, pathological classification as adenocarcinoma, adenosquamous carcinoma, clinical stage IV, peak tricuspid regurgitation velocity, and RV-PA uncoupling (i.e., TAPSE/PASP < 0.67). Cox multivariate regression analysis revealed that clinical stage IV, peak tricuspid regurgitation velocity and RV-PA uncoupling (i.e., TAPSE/PASP < 0.67) were independently correlated with all-cause mortality (p < 0.001).

**Table 6:** Univariable Cox proportional hazard models for all-cause mortality.

Variable	Univariate analysis Hazard ratio (95% CI)	P value
Age	0.994 (0.979–1.010)	0.465
Sex (male)	1.355 (1.047–1.755)	0.021
Current or former smoker	1.305 (1.018–1.672)	0.036
Hypertension	1.024 (0.780–1.344)	0.865
Heart failure	1.682 (1.095–2.583)	0.018
Atrial fibrillation	1.646 (0.730–3.715)	0.230
COPD	1.217 (0.744–1.993)	0.434
Diabetes mellitus	1.031 (0.771–1.379)	0.836
platinum-based drugs and antimetabolites	1.126 (0.842–1.506)	0.422
platinum-based drugs and antimicrotubule drugs	0.917 (0.683–1.230)	0.563
Targeted Drugs	1.367 (0.721–2.592)	0.338
Radiotherapy	1.468 (1.085–1.988)	0.013
Adenocarcinoma	0.701 (0.536–0.918)	0.010
Squamous carcinoma	1.264 (0.941–1.699)	0.120
Adenosquamous	3.197 (1.634–6.255)	0.001
Clinical Stage IV	2.479 (1.838–3.343)	<0.001
peak Tricuspid regurgitation velocity	1.016 (1.013–1.020)	<0.001
Left ventricular ejection fraction (%)	1.004 (0.982–1.027)	0.727
TAPSE/PASP	3.296 (2.477–4.386)	<0.001

Values are presented as median (quartile 1, quartile 3). COPD, chronic obstructive pulmonary disease. TAPSE/PASP, Tricuspid annular plane systolic excursion/pulmonary artery systolic pressure ratio.

**Table 7:** Multivariable Cox proportional hazard models for all-cause mortality.

Variable	Multivariate analysis Hazard ratio (95% CI)	P value
Sex (male)	1.065 (0.718–1.581)	0.754
Current or former smoker	1.247 (0.853–1.824)	0.254
Heart failure	1.341 (0.864–2.081)	0.191
Radiotherapy	1.024 (0.751–1.396)	0.880
Adenocarcinoma	0.942 (0.688–1.289)	0.707
Adenosquamous	1.787 (0.872–3.661)	0.113
Clinical Stage IV	1.757 (1.287–2.399)	<0.001
peak Tricuspid regurgitation velocity	1.011 (1.006–1.015)	<0.001
TAPSE/PASP	1.839 (1.308–2.586)	<0.001

Values are presented as median (quartile 1, quartile 3). TAPSE/PASP, Tricuspid annular plane systolic excursion/pulmonary artery systolic pressure ratio.

## 4 Discussion

Numerous studies have consistently demonstrated that reducing perioperative complications, particularly postoperative concomitant right heart dysfunction, is crucial for improving the survival of NSCLC patients who are undergoing lung resection (4–6). While lung resection is an effective clinical treatment for NSCLC, it is important to consider the close physiological and anatomical relationship between the lungs and the heart. Lung resection can lead to a decrease in the pulmonary vascular bed area, an increase in residual pulmonary blood flow, elevated pulmonary circulatory pressure, and increased right ventricular afterload (16, 17). These factors can contribute to the development of right ventricular dysfunction, which progresses rapidly from compensation to decompensation in comparison to that in the left ventricle. This often creates a vicious cycle that significantly impacts hemodynamics, resulting in impaired body circulation, reduced perfusion pressure to organs and tissues, and ultimately increased patient mortality (9, 10). Our study also found that all patients did not have right ventricular dysfunction before lung resection but developed right ventricular dysfunction after surgery and that cumulative survival was significantly lower in patients with right ventricular dysfunction.

This study is the first to utilize transthoracic echocardiography-derived TAPSE/PASP for the noninvasive evaluation of right ventricular function after lung resection for NSCLC. This study aimed to determine the significance of this assessment in terms of postoperative prognosis. The findings of our study indicate that patients with concurrent right ventricular dysfunction after lung resection for NSCLC have a worse prognosis. Through multivariate Cox regression analysis, it was determined that RV-PA uncoupling (TAPSE/PASP < 0.67) was an independent parameter associated with all-cause mortality. This finding is consistent with the study by Shelley et al. (18), who reported that the presence of right ventricular dysfunction in patients undergoing lung resection was associated with mortality in patients admitted to unplanned intensive care units. Noninvasive assessment of RV-PA coupling (TAPSE/PASP) has been shown to correlate closely with invasive hemodynamics and predict the prognosis in several cardiovascular diseases, such as pulmonary hypertension, heart failure with a reduced ejection fraction, and heart failure with a preserved ejection fraction (15, 19, 20). However, the association between RV-PA coupling and prognosis in patients with NSCLC after lung resection has not been extensively investigated, despite the potential connection of these cardiovascular diseases with lung cancer after lung resection. Tello et al. (15) conducted a study using transthoracic echocardiography-derived TAPSE/PASP < 0.31 mm/mmHg to determine RV-PA uncoupling. The authors found that the TAPSE/PASP was the only echocardiographic metric that was independently correlated with the gold standard for invasive measurements of RV-PA coupling (Ees/Ea). In this cohort of patients with NSCLC after lung resection, 193 (53%) patients exhibited RV-PA uncoupling (TAPSE/PASP ratio < 0.67 mm/mmHg). Notably, the TAPSE/PASP values defining RV-PA uncoupling in the two studies were significantly different, which could be attributed to variations in the study populations. The former study focused on patients with pulmonary hypertension who had an average PASP of 75 ± 24 mmHg and experienced significant damage to the structure and function of the right ventricle.

One of the key findings of this study is that diabetes mellitus is an independent risk factor for right ventricular dysfunction after lung resection in patients with NSCLC. Previous research has demonstrated that diabetes mellitus can impact right ventricular systolic and diastolic function as an independent risk factor and plays a prognostic role in various cardiovascular diseases (21, 22), but there have been no reports on diabetes mellitus being an independent risk factor for concurrent right ventricular dysfunction in patients after lung resection for NSCLC. In a study focusing on patients with pulmonary hypertension, Whitaker et al. (23) discovered a strong association between diabetes mellitus and the severity of pulmonary hypertension, as well as right ventricular wall thickening. They concluded that diabetes mellitus increases vascular stiffness, thereby elevating the afterload on the right ventricle and leading to an increase in right ventricular wall thickness, ultimately resulting in right ventricular dysfunction. In this study, age was identified as a risk factor for developing right ventricular dysfunction in this group of patients, with older patients being more likely to have this dysfunction. This could be attributed to the fact that a significant number of older NSCLC patients had pre-existing chronic lung disease and long-term ventilation dysfunction, leading to an underlying increase in right ventricular afterload (24, 25). Additionally, undergoing lung resection further exacerbates the increase in right ventricular afterload, leading to a decrease in right ventricular function and negatively impacting postoperative functional recovery (26). Li et al. (27) observed an initial decrease in right heart function following radiotherapy for thoracic tumors. Xu et al. (28) reported a significant decrease in 3D RV free wall longitudinal strain (FWLS) after chemotherapy, with the variation in 3D RV FWLS being the sole predictor of subclinical chemotherapy-related cardiac dysfunction (CTRCD). Our study aligns with these findings, indicating that radiotherapy increases the risk of concurrent right ventricular dysfunction in these patients. In this study, we discovered that patients who underwent lung resection for NSCLC and had clinical stage IV disease were more likely to develop right ventricular dysfunction. This could be attributed to the development of pulmonary hypertension in advanced lung cancer patients. Pullamsetti et al. (29) reported that nearly half of the patients in a cohort of 519 individuals with advanced lung cancer exhibited thickening of the pulmonary arteries and pulmonary hypertension. Subsequent studies suggested that the inflammatory response triggered by lung cancer cells may contribute to the development of pulmonary hypertension. Another novel finding of this study is that adenocarcinoma is a risk factor for right ventricular dysfunction after lung resection in patients with NSCLC. This association may be attributed to progressive pulmonary hypertension caused by pulmonary tumor thrombotic microangiopathy (PTTM). These findings align with the literature suggesting that PTTM is primarily linked to poorly differentiated adenocarcinomas (30).

Among all the studies, we consider RV-PA coupling to be a reliable quantitative method for the early assessment of right ventricular function. The cutoff value of 0.67 for the TAPSE/PASP provides a more accurate evaluation of whether right ventricular function is normal or abnormal after lung resection for NSCLC. Additionally, this value holds greater prognostic significance for such patients.

First, the limitation of this study is its single-center retrospective design. Furthermore, echocardiography may not only underestimate PASP but also overestimate PASP. The main reason for the inaccuracy of echocardiographic estimation of PASP is that estimation of PASP using echocardiography requires a two-step process, with each step having its inherent imprecision. The first step is to measure the peak tricuspid regurgitation velocity, which depends on obtaining a good tricuspid regurgitation spectrum. Good tricuspid regurgitation spectroscopy requires accurate measurement of the TR Doppler signal at peak TR velocities and perfect parallel alignment between the Doppler beam and the TR jet. Failure to correct the angle between the Doppler beam and the TR jet can result in an inaccurate determination of the peak TR velocity used to determine the pressure gradient. Even when the TR jet is of sufficient quality and the Doppler beam is aligned optimally, in patients with right heart failure (RHF) and severe tricuspid regurgitation, evaluation of SPAP using TRV is frequently underestimated, due to pronounced enlargement of the effective area of the regurgitating tricuspid orifice, which causes the reduction of TRV and ‘truncation’ of the CW-Doppler spectrum of tricuspid regurgitation (31). The second step is to estimate the RAP using the IVC size and collapsibility. However, the estimate of RAP based on IVC size and its collapsibility is not considered to be accurate and its overestimation is one of the main causes of error in the calculation of SPAP (32, 33). And, echocardiography may also overestimate sPAP in the presence of the following factors: women, arrhythmic cardiac activity, and systemic arterial hypertension (34). Despite the inaccuracy of the echocardiographic estimate of PASP, the correction of right ventricular systolic function by PASP significantly improves the predictive power of the Cox model compared with the independent use of conventional echocardiographic indices of right ventricular systolic function. Finally, it is important to mention that this study assessed only RV-PA coupling at rest. Future research should investigate the role of echocardiography during exercise in revealing RV-PA coupling and to better stratify the risk of patients with RV-PA uncoupling after lung resection for NSCLC.

## 5 Conclusion

After lung resection for NSCLC, the patient’s right ventricular function predicts prognosis, with patients experiencing right ventricular dysfunction having a worse prognosis. It is important to specifically consider diabetes mellitus as an independent risk factor in this population and actively prevent and correct risk factors to reduce the mortality rate.

## Data availability statement

The original contributions presented in the study are included in the article/supplementary material. Further inquiries can be directed to the corresponding authors.

## Ethics statement

The studies involving humans were approved by The Institutional Review Board and Ethics Committee of Chongqing Hospital of Traditional Chinese Medicine. The studies were conducted in accordance with the local legislation and institutional requirements. Written informed consent for participation was not required from the participants or the participants’ legal guardians/next of kin in accordance with the national legislation and institutional requirements.

## Author contributions

XT: Data curation, Software, Writing – original draft. JT: Resources, Visualization, Writing – original draft. QZ: Resources, Software, Writing – original draft. XL: Conceptualization, Supervision, Writing – review & editing. JW: Visualization, Writing – original draft. HS: Formal analysis, Software, Writing – original draft. YZ: Data curation, Investigation, Writing – original draft. SW: Data curation, Investigation, Writing – original draft. JC: Supervision, Writing – review & editing. MW: Funding acquisition, Project administration, Supervision, Writing – review & editing.
